# Association between autologous formalin-fixed tumor vaccine (AFTV) therapy, molecular pathological markers, and survival outcomes in glioblastoma

**DOI:** 10.1007/s10014-025-00507-1

**Published:** 2025-08-07

**Authors:** Shunichi Koriyama, Yoshihiro Muragaki, Masayuki Nitta, Takashi Maruyama, Taiichi Saito, Shunsuke Tsuzuki, Tatsuya Kobayashi, Buntou Ro, Takashi Komori, Kenta Masui, Takakazu Kawamata

**Affiliations:** 1 Department of Neurosurgery, Tokyo Women’s Medical University, Tokyo, Japan; 2 Faculty of Advanced Techno-Surgery (FATS), Institute of Advanced Biomedical Engineering and Science, Graduate School of Medicine, Tokyo Women’s Medical University, Tokyo, Japan; 3 Center for Advanced Medical Engineering Research and Development, Kobe University, Hyogo, Japan; 4 Department of Laboratory Medicine and Pathology, Tokyo Metropolitan Neurological Hospital, Tokyo Metropolitan Hospital Organization, Tokyo, Japan; 5 Department of Pathology, Tokyo Women’s Medical University, Tokyo, Japan

**Keywords:** Glioblastoma, Autologous formalin-fixed tumor vaccine, IDH mutation, p53 expression, Programmed death-ligand 1

## Abstract

Glioblastoma (GBM) is a primary brain tumor, characterized by rapid progression, high recurrence rates, and resistance to standard therapies. Current treatment modalities provide limited survival benefits, highlighting the need for novel therapeutic strategies. This retrospective study evaluated the efficacy of autologous formalin-fixed tumor vaccine (AFTV) in 375 patients with newly diagnosed GBM. Patients receiving AFTV therapy (n = 164) showed significantly improved progression-free survival (PFS; 14.0 months vs. 8.7 months, *p* = 0.03) and overall survival (OS; 32.0 months vs. 21.9 months, *p* < 0.01) compared with the non-AFTV group (n = 211). Subgroup analyses revealed that AFTV therapy was particularly effective in patients with wild-type IDH tumors and those negative for PD-L1 and p53 expression. In contrast, patients whose tumors were positive for both PD-L1 and p53 exhibited significantly poorer outcomes. These findings suggest that the combination of PD-L1 and p53 status may serve as a useful biomarker for predicting AFTV responsiveness, reflecting the influence of the immunosuppressive tumor microenvironment on treatment efficacy. These findings establish AFTV as a promising treatment option for GBM and highlight the importance of molecular profiling in treatment selection. Future studies should explore combining AFTV with immune checkpoint inhibitors to enhance efficacy in PD-L1-positive cases.

## Background

Glioblastoma (GBM) is the most aggressive type of primary brain tumor in adults, and is classified as a World Health Organization (WHO) Grade IV tumor. It is characterized by rapid progression and a high recurrence rate. Standard treatment involves maximal tumor resection, radiotherapy, and concomitant chemotherapy with temozolomide (TMZ). However, despite advancements in standard therapies, the 5-year survival rate remains < 10% [1, 2]. Furthermore, even in cases where gross total resection (GTR) is achieved, the outcomes remain poor, with a reported median overall survival (OS) ranging from 15 to 20 months [3–5]. These findings highlight the urgent need for novel adjuvant therapies to improve survival outcomes.

Advances in genetic analysis technologies have significantly enhanced our understanding of the molecular genetics and epigenetic characteristics of GBM [6, 7]. Key diagnostic markers include IDH1/2 mutations, TERT promoter mutations, EGFR alterations, and chromosomal anomalies such as 7 gain/10 loss, which are critical components of the WHO classification [8, 9]. Despite these advances, translating these findings into curative treatment strategies remains challenging. Overcoming the resistance of GBM to treatment requires the development of novel therapeutic approaches based on these findings. 

Immunotherapy is a promising treatment option for GBM. Immunotherapy aims to eliminate malignant cells by inducing immune responses against tumor-specific antigens [10]. Among these approaches, autologous formalin-fixed tumor vaccine (AFTV), which we studied and reported in our clinical trial, represents a novel strategy. AFTV is prepared by formalin-fixing the tumor tissue of a patient, presenting tumor-specific antigens to the immune system, and eliciting a targeted immune response. In phase I/II trials, combining AFTV with radiotherapy and TMZ resulted in a median OS of 19.8 months, with the 2-year survival rate being 40% [11, 12]. Furthermore, phase IIb trials demonstrated that in patients who underwent gross total resection (GTR), the 3-year OS reached 80%, while the 3-year progression-free survival (PFS) was 81% in the AFTV group. These results suggested that AFTV therapy has the potential to improve long-term outcomes in patients with GBM [13]. These findings highlight the potential of AFTV to fill a critical gap in GBM therapy by providing a safe and effective adjunctive treatment option.

This study evaluated the clinical outcomes of AFTV therapy in patients with newly diagnosed GBM, focusing on its relationship with key molecular pathological markers, such as IDH mutations, p53 alterations, and Programmed Death-Ligand 1 (PD-L1) expression. By elucidating these associations, we aimed to optimize AFTV therapy and contribute to the development of personalized treatment strategies to improve patient outcomes and address GBM treatment resistance.

## Methods

### Patient selection

A total of 447 patients diagnosed with GBM were included in this retrospective cohort study from Tokyo Women’s Medical University between 2006 and 2021. GBM diagnosis was confirmed through pathological evaluation following the WHO diagnostic criteria available at the time, prior to the 2021 update, and was determined independently of IDH mutation status. The study population was divided into two groups: 164 patients were treated with AFTV therapy in addition to standard treatment (AFTV group), whereas 211 patients were treated with standard treatment alone (non-AFTV group). Standard treatment was comprised of maximal tumor resection, radiotherapy, and TMZ-based chemotherapy.

### Treatment protocol

Maximal tumor resection was performed on all patients with emphasis placed on gross total resection (GTR). Intraoperative MRI (iMRI) was employed during surgery to optimize the extent of tumor resection and minimize damage to surrounding functional areas [14–16]. In addition, intraoperative flow cytometry (iFC) was utilized for the rapid intraoperative assessment of tumor cellularity and margin diagnosis, enabling precise determination of tumor borders during resection [17–19].

In selected cases, photodynamic therapy (PDT) was implemented as an adjunct to surgery. A photosensitizing agent was administered and subsequently activated using a specific wavelength of light, inducing localized cytotoxic effects. Residual tumor cells were targeted in areas where complete resection was challenged by proximity to critical structures. The PDT-induced enhancement of tumor control and potential prolongation of survival in patients with GBM has been demonstrated when integrated with standard surgical and adjuvant treatments [20, 21].

Postoperative adjuvant therapy was administered to all patients and was comprised of radiotherapy and TMZ-based chemotherapy according to standard protocols. In the AFTV group, AFTV was administered subcutaneously at 2-week intervals for a total of three doses. AFTV was prepared by formalin fixation of viable portions of the resected tumor tissue of the patient, whereby tumor-specific antigens were preserved and a targeted immune response was induced [11–13].

### Outcomes and subgroup analysis

Clinical outcomes were evaluated, specifically PFS and OS. Survival was measured from the date of surgery to the date of tumor recurrence, progression, or death. Subgroup analyses were performed to investigate the potential effect of AFTV therapy. The following analyses were conducted:

1. Survival outcomes were evaluated in patients aged ≥ 65 years by comparing the AFTV and non-AFTV groups.
2. OS was examined in patients for whom early recurrence was documented within 6 months of the initial treatment.

### Molecular pathological analysis

*Resected tumor tissue* was analyzed for key molecular pathological markers. IDH1/2 mutations were assessed using IDH1 R132H-specific immunohistochemistry (IHC). For samples in which staining was not observed, additional analysis was performed using PCR, targeting the R132 hotspot mutation in IDH1 and R172 hotspot mutation in IDH2. The expression of the p53 protein was assessed using IHC, and the mutation status was inferred based on the staining intensity.

PD-L1 expression was evaluated using IHC, whereby assessments were based on the intensity and extent of membrane staining in tumor cells. Staining was performed using a rabbit monoclonal antibody against PD-L1 (clone E1L3N^®^, Cell Signaling Technology, #13684), at a dilution of 1:200, following the manufacturer’s recommended protocol. Cases were considered PD-L1 positive when ≥ 25% of tumor cells exhibited membrane staining of any intensity [22]. Necrotic areas were excluded from evaluation due to the likelihood of nonspecific staining. This assessment criterion was adopted based on existing literature and clinically validated methodologies.

In addition to PD-L1 analysis, immune cell infiltration was assessed, including that of CD3-positive T-cells, CD4-positive helper T-cells, and CD8-positive cytotoxic T-cells. A comprehensive investigation of the relationship between molecular pathological characteristics of tumors and the immune microenvironment was thereby enabled. Due to limitations in tissue quality and quantity, all analyses could not be performed on every sample.

### Statistical analysis

Kaplan–Meier survival curves were generated for estimation of PFS and OS in the AFTV and non-AFTV groups. Differences in survival between the groups were assessed using the log-rank test. Statistical analyses were performed using the R software (version 4.0.3; Supplier, city, [state], country), and statistical significance was defined as *p* < 0.05.

## Results

### Patient characteristics

Of 477 newly diagnosed patients with GBM treated at Tokyo Women’s Medical University between 2006 and 2021, 416 patients who received the Stupp regimen and had sufficient follow-up data were included in this analysis. After excluding 41 biopsy cases, 375 patients who underwent maximal resection remained for evaluation.

The study comprised 164 patients in the AFTV group and 211 in the non-AFTV group. The median age was significantly lower in the AFTV group (51.2 ± 14.4 years) than in the non-AFTV group (56.1 ± 15.1 years; *p* < 0.01). Although the AFTV group had a significantly lower median age, the therapeutic effects of AFTV therapy in elderly patients were analyzed as part of our study design to assess its efficacy across different age groups and account for potential age-related biases. No statistically significant differences were observed between the two groups in terms of sex ratio, Karnofsky Performance Status (KPS), laterality, or tumor location. The baseline characteristics of the patients are summarized in Table 1.

**Table 1:** Baseline characteristics of the study population

Variable	AFTV Group (n = 164)	non-AFTV Group (n = 211)	*p*-value
Sex (M/F)	103/61	116/95	*p* = 0.16
Age (years)	50.6 ± 14.6	55.8 ± 15.4	*p* < 0.01
Laterality (R/L)	53/48	50/52	*p* = 0.25
Location	Temporal 36.4%	Frontal 40.0%	*p* = 0.26
	Frontal 31.8%	Temporal 23.5%	
	Parietal 19.6%	Parietal 15.7%	
	Other 12.1%	Other 20.9%	
*Pre-operative KPS*			
≥ 80	64.8%	50.0%	*p* = 0.40
≤ 70	35.2%	50.0%	
Tumor size (mm)	51.4 ± 17.2	45.3 ± 15.7	*p* < 0.01

### Overall efficacy of AFTV therapy

**Fig. 1:**
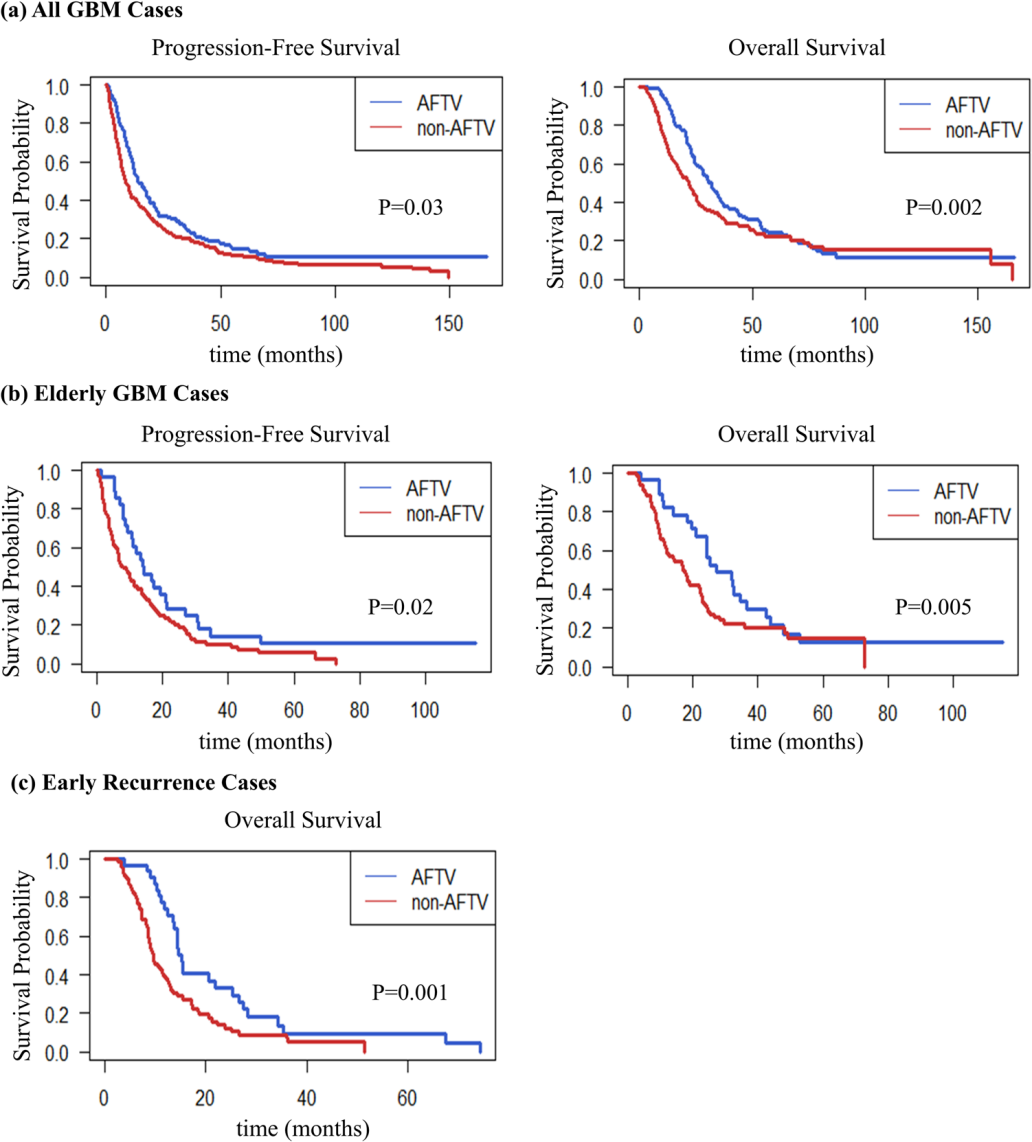
Kaplan–Meier survival curves illustrating the efficacy of AFTV therapy across various subgroups. **a** All Patients with GBM: AFTV therapy significantly improved survival outcomes compared to the non-AFTV group. The median progression-free survival (PFS) was 14.0 months in the AFTV group and 8.7 months in the non-AFTV group (*p* = 0.03). The median overall survival (OS) was 32.0 months in the AFTV group and 21.9 months in the non-AFTV group (*p* = 0.002). **b** Elderly GBM Cases: Among patients aged 65 years or older, AFTV therapy significantly extended survival. The median PFS was 14.2 months in the AFTV group and 8.1 months in the non-AFTV group (*p* = 0.02), while the median OS was 27.3 months in the AFTV group and 17.0 months in the non-AFTV group (*p* = 0.005). **c** Early Recurrent Cases: In cases of recurrence within six months of initial treatment, AFTV therapy led to significantly prolonged survival. The median OS was 15.1 months in the AFTV group and 9.5 months in the non-AFTV group (*p* = 0.001)

Comparison between the AFTV (n = 164) and non-AFTV (n = 211) groups demonstrated significantly prolonged PFS and OS in the AFTV group. The median PFS was 14.0 months in the AFTV group versus 8.7 months in the non-AFTV group (*p* = 0.03; Fig. 1a). Similarly, the median OS was 32.0 months in the AFTV group compared with 21.9 months in the non-AFTV group, demonstrating a statistically significant improvement (*p* < 0.01; Fig. 1a). These results suggest the potential of AFTV therapy to improve the prognosis of patients with GBM.

### Illustrative case 1

**Fig. 2:**
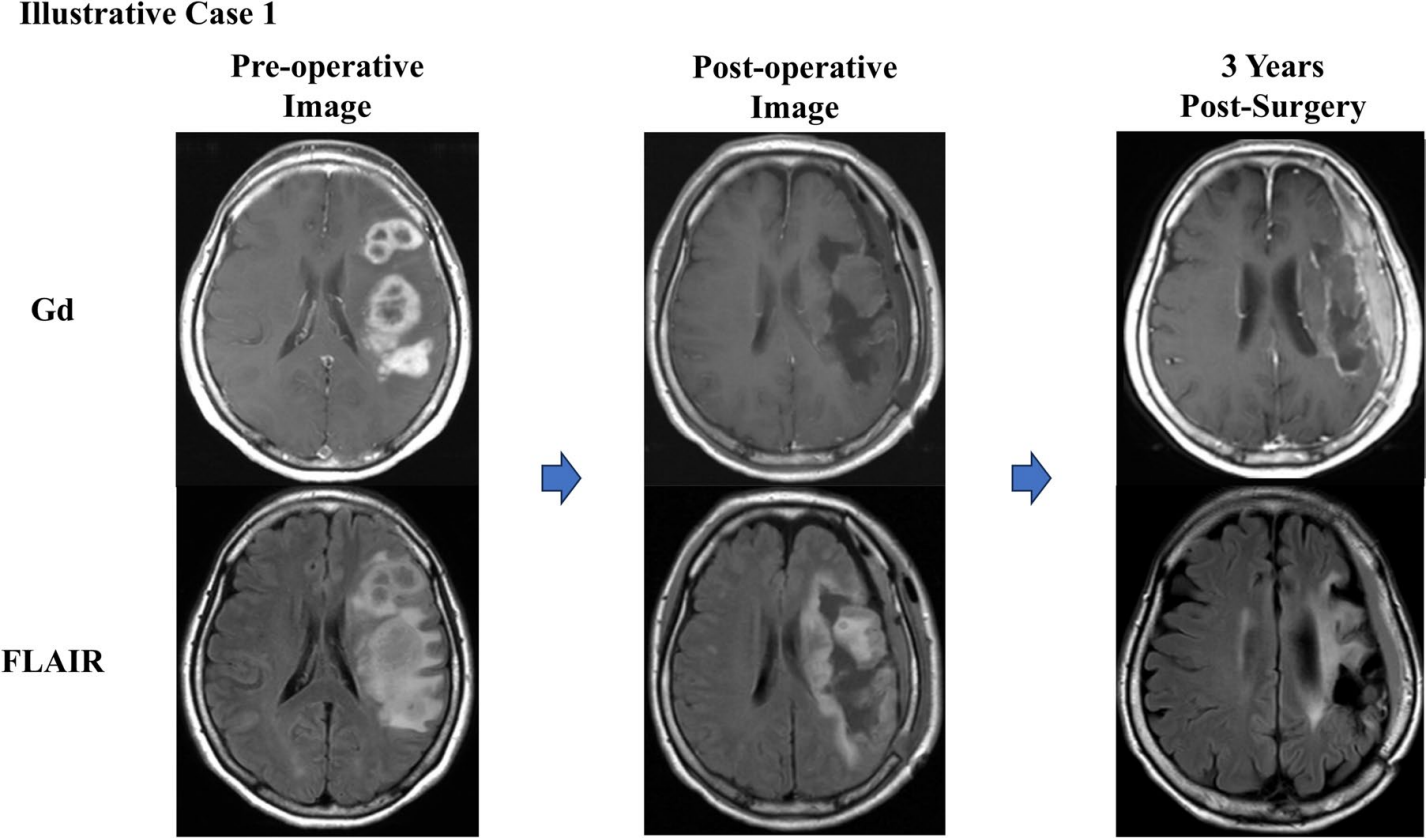
Illustrative Case 1, a 62-year-old female with glioblastoma (GBM) who achieved long-term disease control with AFTV therapy. Preoperative MRI revealed multiple contrast-enhancing lesions in the left frontal and parietal lobes. The patient underwent gross total resection (GTR) combined with photodynamic therapy (PDT), followed by AFTV therapy. Postoperative MRI confirmed successful lesion removal, and at nine years post-surgery, no recurrence has been observed. Despite moderate aphasia and mild hemiparesis, the patient continues to maintain disease control, highlighting the potential of AFTV therapy in achieving durable remission

A 62-year-old woman presented with progressive aphasia and right hemiparesis. MRI revealed multiple contrast-enhancing lesions in the left frontal and parietal lobes. The patient underwent tumor removal and photodynamic therapy (PDT) to achieve gross total resection (GTR) of the enhancing lesions. Postoperatively, AFTV therapy was administered. Despite moderate aphasia and mild hemiparesis, the patient has maintained a Karnofsky Performance Status (KPS) of 60 and has remained recurrence-free for 9 years postoperatively. This case highlights the potential of AFTV therapy in achieving exceptional long-term disease control (Fig. 2).

#### Efficacy of AFTV in older patients

We evaluated the efficacy of AFTV therapy in 113 older patients (aged ≥ 65 years), including 29 in the AFTV group and 82 in the non-AFTV group. The median PFS was 14.2 months in the AFTV group compared with 8.1 months in the non-AFTV group (*p* = 0.02; Fig. 1b). Similarly, the median OS was 27.3 months in the AFTV group compared with 17.0 months in the non-AFTV group, demonstrating a statistically significant improvement (*p* < 0.01; Fig. 1b). These results demonstrated that AFTV therapy is a viable treatment option for older patients with GBM, who typically have poor prognoses.

#### Efficacy of AFTV in early recurrence cases

Among 114 early recurrence cases (defined as recurrence within 6 months of initial treatment), 32 patients were in the AFTV group and 82 were in the non-AFTV group. The median OS was 15.1 months in the AFTV group compared with 9.5 months in the non-AFTV group (*p* < 0.01; Fig. 1c). These findings indicated that AFTV remains effective even in cases of aggressive disease progression.

### Illustrative case 2

**Fig. 3:**
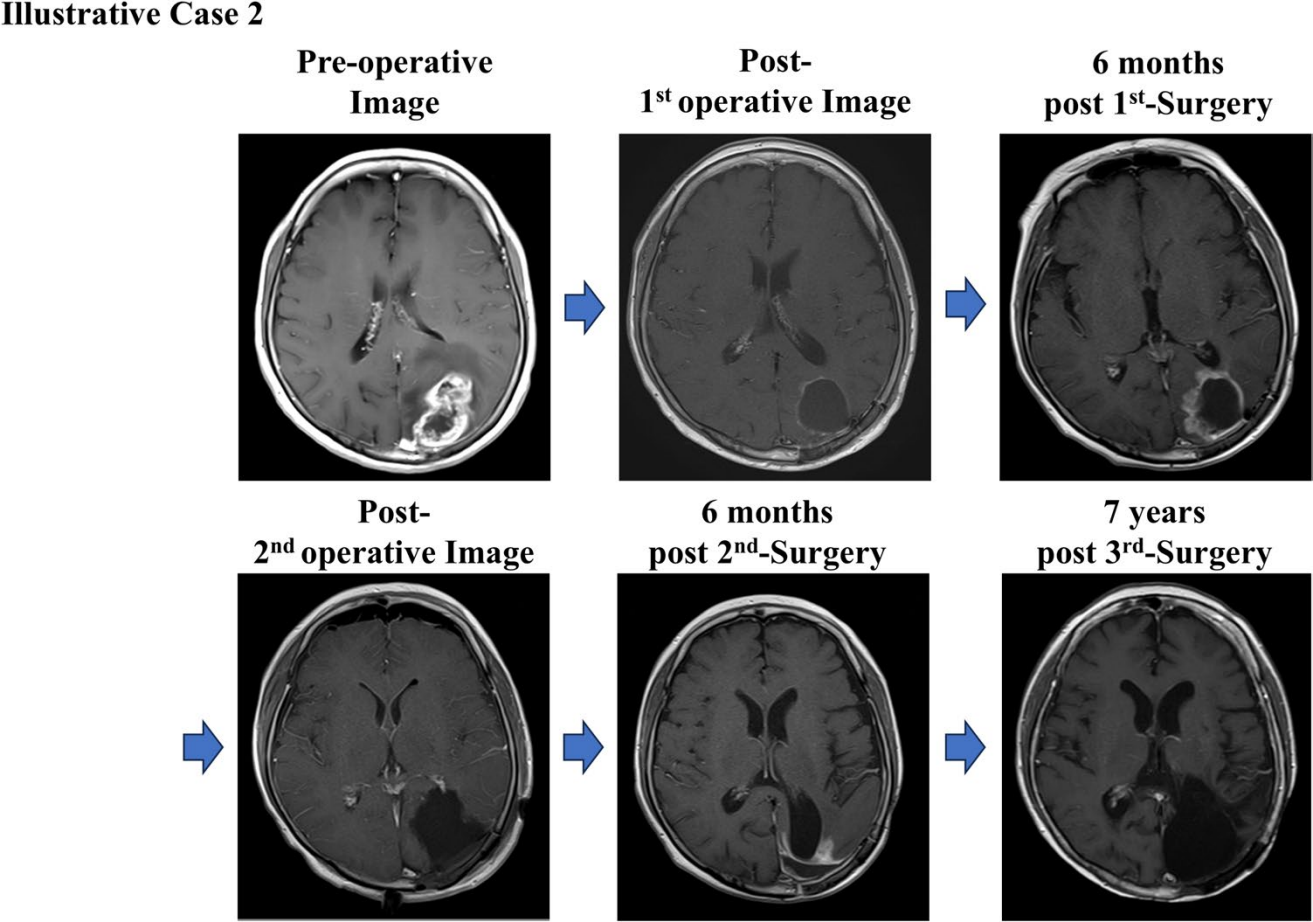
Illustrative Case 2, a 59-year-old female with early recurrent glioblastoma (GBM) who achieved long-term survival. Preoperative MRI revealed a contrast-enhancing lesion in the left occipital lobe. After gross total resection, she received radiotherapy, chemotherapy, and AFTV therapy. Tumor recurrence occurred twice within six months of the previous surgery, requiring two additional resections with photodynamic therapy (PDT). The final pathology showed predominantly necrotic tissue with minimal tumor cells. The patient has remained recurrence-free for seven years following the third surgery, achieving a total survival of eight years with a Karnofsky Performance Status (KPS) of 80. This case suggests a potential delayed effect of AFTV therapy, even in early recurrent GBM

A 59-year-old woman presented with right hemianopia; MRI revealed a contrast-enhancing lesion in the left occipital lobe. Following gross total resection at another institution, the patient was referred to our hospital, where radiotherapy, chemotherapy, and AFTV therapy were initiated. Six months after treatment, recurrence was observed along the resection cavity wall. The patient underwent a second tumor resection with PDT, and pathological analysis confirmed recurrent GBM. Six months later, a second recurrence occurred, and another resection was performed. Pathological examination of the specimen revealed predominantly necrotic tissue with minimal tumor cells. The patient has remained recurrence-free for seven years, achieving a total survival of eight years from initial surgery, with a maintained KPS of 80. This case suggested a potentially delayed efficacy of AFTV therapy, even in cases of early recurrence (Fig. 3).

### Association between molecular pathological factors and AFTV therapy

#### IDH mutation and AFTV efficacy

**Fig. 4:**
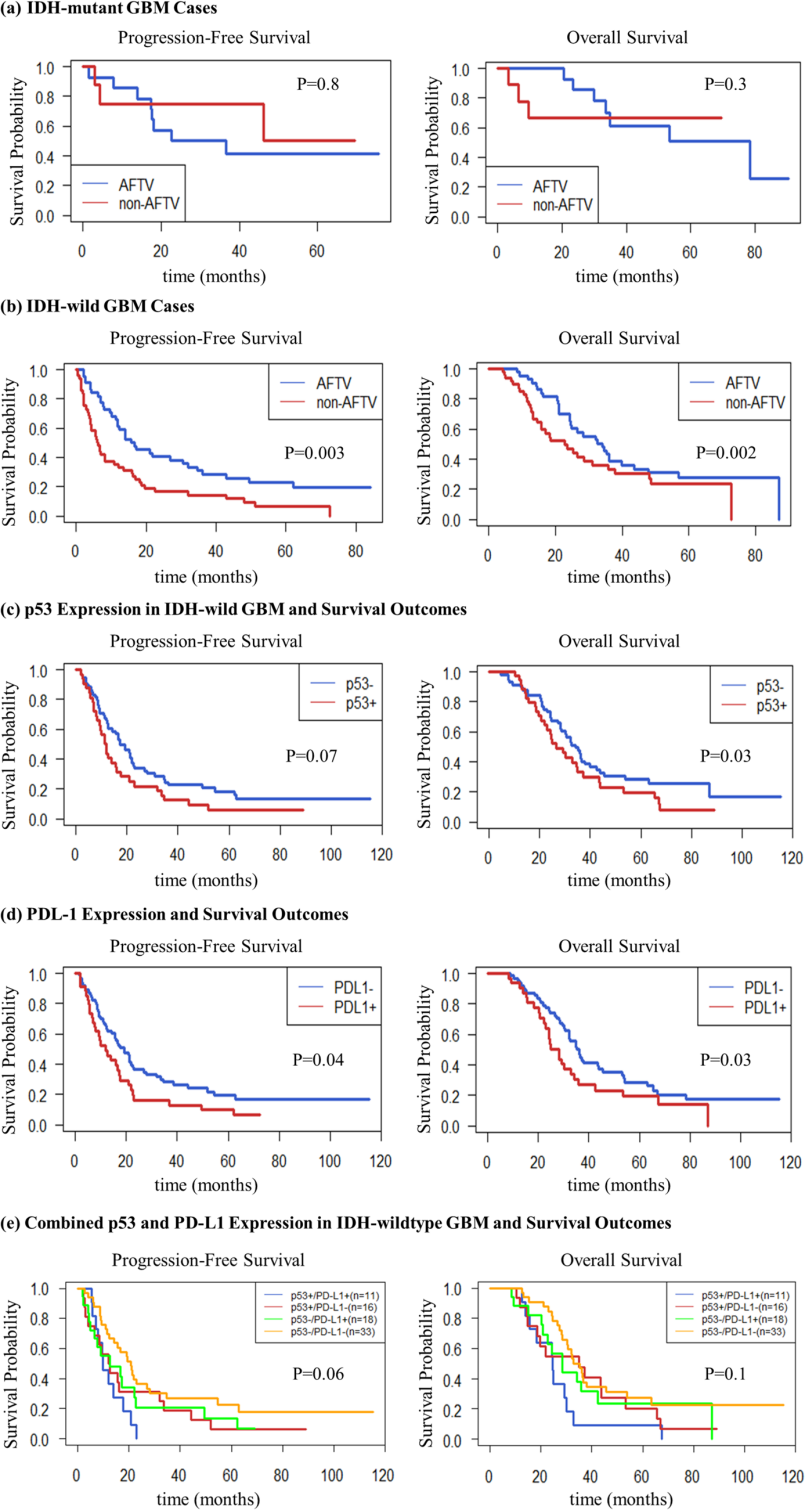
Kaplan–Meier survival curves illustrating the association between molecular pathological markers and AFTV therapy. **a** IDH-mutant GBM cases: No significant differences in progression-free survival (PFS, *p* = 0.8) or overall survival (OS, *p* = 0.3) were observed between the AFTV and non-AFTV groups, suggesting limited efficacy of AFTV therapy in IDH-mutant GBM. **b** IDH wild-type GBM cases: AFTV therapy significantly improved survival outcomes. The median PFS was 16.2 months in the AFTV group versus 6.3 months in the non-AFTV group (*p* = 0.003), and the median OS was 34.2 months versus 22.9 months, respectively (*p* = 0.002). **c** p53 expression in IDH wild-type GBM: Patients with p53-negative tumors showed significantly longer OS compared with those with *p53*-positive tumors (35.3 vs 24.8 months, *p* = 0.03). PFS also tended to be longer in the *p53*-negative group (17.2 vs 11.6 months, *p* = 0.07). **d** PD-L1 expression and survival outcomes: AFTV therapy was more effective in PD-L1-negative cases, with a median PFS of 19.1 months versus 9.8 months in PD-L1-positive cases (*p* = 0.04), and a median OS of 36.1 months versus 28.3 months, respectively (*p* = 0.03). **e** Combined p53 and PD-L1 status in IDH wild-type GBM: Patients with tumors negative for both p53 and PD-L1 (double-negative) had the most favorable prognosis, while those with tumors positive for both markers (double-positive) had the poorest. Median PFS was 20.9 months in the double-negative group versus 9.8 months in the double-positive group (*p* = 0.04), and median OS was 35.3 months versus 24.5 months, respectively (*p* = 0.03). A log-rank test for overall comparison among the four subgroups revealed a borderline difference in PFS (*p* = 0.06) and a similar trend in OS (*p* = 0.1)

We evaluated the association between IDH status and AFTV efficacy in 115 patients, consisting of 22 patients with IDH-mutant and 93 with wild-type IDH tumors. Among the 22 patients with IDH-mutant tumors (14 in the AFTV group and 8 in the non-AFTV group), we did not detect any significant differences in PFS (*p* = 0.8) or OS (*p* = 0.3) between the groups. These findings suggested that AFTV therapy has limited efficacy in altering the outcomes of IDH-mutant GBM, which is associated with a favorable prognosis (Fig. 4a).

Among the 93 patients with wild-type IDH (44 in the AFTV group and 49 in the non-AFTV group), we identified significant improvements in the survival outcomes of those treated with AFTV. The median PFS was 16.2 months in the AFTV group, which was substantially longer than the 6.3 months observed in the non-AFTV group (*p* < 0.01). Similarly, the median OS was 34.2 months in the AFTV group compared with 22.9 months in the non-AFTV group, showing a statistically significant improvement (*p* < 0.01; Fig. 4b). These findings indicated the efficacy of AFTV therapy in improving the survival outcomes of patients with wild-type IDH GBM.

### Association between p53 expression and AFTV therapy in wild-type IDH GBM

We evaluated the prognostic effect of p53 expression on AFTV therapy efficacy in 91 patients with wild-type IDH GBM, including 59 in the AFTV group and 32 in the non-AFTV group. In the p53-negative subgroup, patients exhibited longer PFS and OS compared to those in the p53-positive subgroup. The median PFS was 17.2 months in the p53-negative subgroup and 11.6 months in the p53-positive subgroup (*p* = 0.07). The median OS was 35.3 months in the p53-negative subgroup and 24.8 months in the p53-positive subgroup, showing a statistically significant improvement (*p* = 0.03) (Fig. 4c).

### PD-L1 expression and AFTV efficacy

We conducted PD-L1 expression analysis in 97 patients, 63 of whom were classified as PD-L1-negative, whereas 34 were classified as PD-L1-positive.

In PD-L1-negative patients, the median PFS was 19.1 months, which was significantly longer than the 9.8 months observed in PD-L1-positive patients (*p* = 0.04). Similarly, the median OS was 36.1 months in PD-L1-negative patients compared with 28.3 months in PD-L1-positive patients, demonstrating a statistically significant difference (*p* = 0.03). These findings suggested the enhanced efficacy of AFTV therapy in patients with PD-L1-negative disease. Nonetheless, even in PD-L1-positive patients, AFTV therapy contributed to improved prognosis compared with that in untreated patients, albeit with reduced efficacy, suggesting that PD-L1 status may influence the therapeutic response to AFTV.

### Combined p53 and PD-L1 status and AFTV efficacy

We further classified 78 patients with wild-type IDH GBM who had complete p53 and PD-L1 expression data into four subgroups based on their p53 and PD-L1 immunostaining status: p53 + /PD-L1 + , p53 + /PD-L1 − , p53 − /PD-L1 + , and p53 − /PD-L1 − . A log-rank test for overall comparison among the four groups revealed a borderline difference in PFS (*p* = 0.06) and a similar trend in OS (*p* = 0.1) (Fig. 4e). Notably, direct comparison of the p53 − /PD-L1 − subgroup, which showed the most favorable outcome, and the p53 + /PD-L1 + subgroup, which showed the poorest, demonstrated significant differences in survival. The median PFS was 20.9 months in the p53 − /PD-L1 − subgroup and 9.8 months in the p53 + /PD-L1 + subgroup (*p* = 0.04). The median OS was 35.3 months in the p53 − /PD-L1 − subgroup and 24.5 months in the p53 + /PD-L1 + subgroup (*p* = 0.03), suggesting that the absence of both p53 overexpression and PD-L1 expression is associated with improved therapeutic response to AFTV.

### Evaluation of the immune microenvironment

We also evaluated the immune microenvironment in both PD-L1-positive (n = 34) and PD-L1-negative (n = 63) groups. We found that CD3-positive T-cells and CD8-positive cytotoxic T-cells infiltrated the peritumoral area in both groups. CD4-positive helper T-cells were scarce, with 22.7% (5/22) of patients in the PD-L1-positive group and 5.4% (2/37) in the PD-L1-negative group (*p* = 0.09). CD8-positive cytotoxic T-cell infiltration was comparable between the groups and was observed in 95.5% (21/22) of PD-L1-positive and 94.6% (35/37) of PD-L1-negative (*p* = 1.00) patients. Macrophage infiltration (CD68-positive) was confirmed in all cases regardless of PD-L1 expression.

**Fig. 5:**
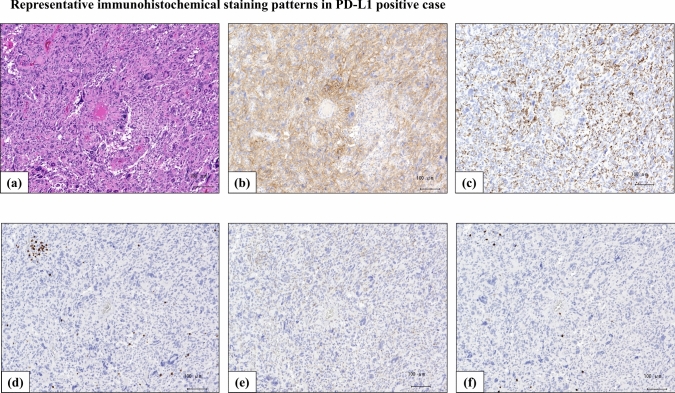
Representative immunohistochemical staining patterns in a PD-L1-positive glioblastoma case. **a** Hematoxylin and eosin (H&E) staining showing characteristic glioblastoma histopathology. **b** PD-L1 immunohistochemistry demonstrating strong membranous positivity. **c** CD68 immunostaining confirming macrophage infiltration. **d** CD3 immunostaining indicating T-cell infiltration. **e** CD4 immunostaining showing a lack of helper T-cell activity. **f** CD8 immunostaining demonstrating cytotoxic T-cell infiltration in the peritumoral area. CD3-positive and CD8-positive T cells were present, while CD4-positive helper T cells were rarely observed. A similar distribution of immune cells was noted in PD-L1-negative cases

The distribution of immune cells in PD-L1-positive tumors showed a trend toward increased CD4-positive cells but did not differ significantly from that in PD-L1-negative tumors. Figure 5 shows representative immunohistochemical staining patterns of PD-L1-positive tumors.

## Discussion

### Challenges in glioblastoma (GBM) and the potential of AFTV therapy

GBM, one of the most challenging malignancies of the central nervous system, is characterized by rapid progression, high recurrence rates, and limited treatment options. Immunotherapies, including AFTV therapy, have emerged as promising strategies for improving patient outcomes. This study demonstrated that AFTV therapy contributes to prolonged PFS and OS, and elucidated the differential effect of molecular pathological factors, such as IDH mutations, p53 expression, and PD-L1 expression, on treatment efficacy.

### Efficacy and potential of AFTV therapy

Our study confirmed that AFTV therapy significantly prolonged OS in patients with GBM. The median OS in the AFTV group was 32.0 months, which was significantly longer than the 21.9 months observed in the non-AFTV group (*p* < 0.01). These results highlight the potential of AFTV therapy as a promising treatment strategy for GBM.

Notably, AFTV has demonstrated efficacy, even in patients with traditionally poor prognoses. Among older patients (aged ≥ 65 years), the median OS in the AFTV group was 27.3 months, significantly exceeding the 17.0 months observed in the non-AFTV group (*p* < 0.01). Older patients with GBM often have limited treatment options because of their reduced tolerance to standard therapies, including chemotherapy and other cancer treatments [23–26]. Hence, AFTV therapy, with its favorable safety profile, is a viable alternative treatment for this vulnerable population.

In addition, AFTV therapy showed efficacy in early recurrence cases, defined as recurrence within 6 months of the initial treatment. This patient group is typically associated with extremely poor prognoses; however, the median OS was 15.1 months in the AFTV group versus 9.5 months in the non-AFTV group (*p* < 0.01). These findings suggested that AFTV therapy induced sustained immune responses that contributed to therapeutic efficacy even in recurrent cases. For example, a patient (Case 2) achieved long-term survival despite two recurrences, suggesting a delayed-onset mechanism of action for AFTV therapy.

### Molecular pathological factors and the efficacy of AFTV therapy

#### IDH mutation and AFTV efficacy

In wild-type IDH GBM, AFTV therapy significantly improved survival outcomes (PFS: 16.2 months versus 6.3 months, OS: 34.2 months versus 22.9 months; *p* < 0.01). This finding aligned with previous research indicating that wild-type IDH tumors, characterized by higher neoantigen loads, are more likely to elicit robust immune responses. In contrast, AFTV therapy did not provide significant survival benefits in patients with IDH-mutant GBM. IDH mutations induce metabolic changes and promote the production of 2-hydroxyglutarate, contributing to the formation of an immunosuppressive tumor microenvironment [27–30].

### p53 expression and AFTV efficacy

AFTV therapy showed significantly greater efficacy in p53-negative cases, particularly with respect to overall survival (*p* = 0.03), while progression-free survival showed a favorable trend (*p* = 0.07). These results suggest that intact p53 function supports an effective immune response to AFTV therapy [13]. Conversely, in patients with p53-positive tumors demonstrated significantly shorter survival, indicating that p53 mutations may contribute to the formation of an immunosuppressive tumor microenvironment. Previous studies have shown that p53 mutations promote the recruitment of regulatory T-cells (Tregs), whereas suppress effector T-cell activity [31, 32]. These findings supported the hypothesis that p53 mutations attenuate the therapeutic efficacy of AFTV.

### PD-L1 expression and immune evasion mechanisms

In this study, AFTV therapy demonstrated significantly greater efficacy in patients with PD-L1-negative tumors (PFS: 19.1 months versus 9.8 months, *p* = 0.04; OS: 36.1 months versus 28.3 months, *p* = 0.03), whereas its efficacy diminished in those with PD-L1-positive tumors. These results suggested that PD-L1 plays a critical role in immune evasion mechanisms, which may limit the effectiveness of AFTV therapy.

Immune microenvironment analysis revealed the presence of CD3-positive and CD8-positive T-cells around the tumor, regardless of PD-L1 expression. However, CD4-positive helper T-cells were scarce in both groups, with a trend toward increased numbers of CD4-positive cells in PD-L1-positive tumors. This trend may suggest a potential involvement of regulatory T-cells (Tregs), which warrants further investigation. Previous studies have reported increased Treg infiltration in PD-L1-positive tumors, which enhances immune suppression and facilitates immune evasion. This immunosuppressive tumor microenvironment may partly explain the reduced efficacy of AFTV therapy in PD-L1-positive tumors [32–34].

Based on these findings, combining AFTV therapy with immune checkpoint inhibitors targeting PD-L1 could potentially improve the therapeutic outcomes in patients with PD-L1-positive tumors. Such an approach may help overcome Treg-mediated immunosuppression and enhance effector T-cell activity [32, 33].

Furthermore, when combining p53 and PD-L1 expression statuses, we observed significantly better outcomes in patients whose tumors were negative for both markers. Specifically, the p53 − /PD-L1 − subgroup showed the longest median PFS and OS, whereas the p53 + /PD-L1 + subgroup exhibited the poorest outcomes. These results suggest that co-occurring p53 overexpression and PD-L1 expression may synergistically contribute to immune resistance and attenuate the therapeutic efficacy of AFTV. The combined assessment of these molecular markers could serve as a useful predictor for AFTV responsiveness and may help identify patients most likely to benefit from this therapy.

## Conclusion

AFTV therapy improved OS in patients with GBM, including those with previously poor prognoses such as older patients, early recurrence cases, and wild-type IDH tumors. In addition, the efficacy of AFTV was significantly influenced by molecular pathological factors such as PD-L1 expression and p53 status. Immune evasion mechanisms associated with PD-L1 expression appeared to limit the efficacy of AFTV therapy, and patients whose tumors exhibited both PD-L1 expression and p53 overexpression showed the poorest prognosis. These findings suggest that the combination of PD-L1 and p53 status may serve as a useful biomarker to predict AFTV responsiveness.

Further clinical trials, including ongoing physician-initiated phase III studies, are essential to validate these findings and establish strategies to overcome immune evasion associated with IDH mutations, p53 mutations, and PD-L1 expression. Combining AFTV therapy with immune checkpoint inhibitors has the potential to enhance therapeutic outcomes in patients with PD-L1-positive GBM.

This study indicated the transformative potential of AFTV therapy for GBM treatment and highlighted the importance of personalized approaches based on molecular pathological characteristics.

## Data Availability

The data supporting the findings of this study are not publicly available due to ethical and privacy concerns but may be shared by the corresponding author upon reasonable request.
